# Non-Destructive Analytical Investigation of Decorative Wallpapers Samples of the Nineteenth Century before Their Restoration

**DOI:** 10.3390/s21134416

**Published:** 2021-06-28

**Authors:** Ilaria Costantini, Kepa Castro, Maria Dolores Rodriguez-Laso, Juan Manuel Madariaga, Gorka Arana

**Affiliations:** 1 Department of Analytical Chemistry, Faculty of Science and Technology, University of the Basque Country UPV/EHU, P.O. Box 644, 48080 Bilbao, Basque Country, Spain; ilaria.costantini@ehu.eus (I.C.); kepa.castro@ehu.eus (K.C.); gorka.arana@ehu.eus (G.A.); 2 Department of Painting, University of the Basque Country, P.O. Box 644, 48080 Bilbao, Spain; mariadolores.rodriguez@ehu.eus

**Keywords:** decorative wallpapers, raman spectroscopy, micro-EDXRF, ATR-FTIR, pigments, cellulose

## Abstract

In this work, decorative wallpapers (19th century) from an historical palace located in Oiartzun (Basque Country, Spain) were analyzed before their restoration. Micro-energy dispersive X-ray fluorescence spectroscopy, Raman spectroscopy, and attenuated total reflectance infrared spectroscopy were used to investigate the elemental and molecular composition of pigments, the presence of binders, and the state of conservation of the paper support. The aim of the investigation was trying to understand the possible degradation pathways and identify the raw materials in order to choose the best restoration protocol according to the original aspect of wallpapers. As stated from both the elemental distribution and the identification of mineral phases by Raman spectroscopy, the most used pigment was lead chromate. It was mixed with other pigments such as ultramarine blue, zinc chromate, hematite, and atacamite among others to obtain different shades and they were applied mixed with an animal glue. Brass, identified thanks to elemental micro-EDXRF maps, was employed as a shiny decorative element. In addition, a partial degradation of cellulose was detected due to its natural ageing, the acidic nature of lignin, and to a phenomenon of humidity of the walls. Probably the deposition of black particulate matter was the cause of the darkening of the painting surfaces.

## 1. Introduction

Artworks on paper such as manuscripts, lithographs, watercolors, etc., are one of the most sensitive categories of artworks especially when they are exposed in open spaces, like decorative wallpapers, and they need special attention to be preserved. The importance of the study of wallpapers has received more attention over the past decades as a result of the increasing interest of conservation of private cultural heritage preserved in the interior of historical houses and palaces. Private heritage has been converted into public heritage and many historic houses have been adapted to museums or art galleries. 

The origins of the manufacture of wallpaper can be located in the East from where they arrived in Europe through the commercial routes. At the end of the 17th century, European manufacturers were already developing paper as a wallcovering. Gradually, it became an essential decorative element in the homes of the bourgeois, for which a great interest quickly arose in making these products avoiding import from the East [1].

At the end of the 18th century, the manufacture of wallpapers reached a maximum in production. France, England, Germany, Switzerland, and Belgium were some of the leading countries in the creation and production of decorative wallpapers. In this period, some important factories were founded in Europe, such as the one in Rixheim by Juan Zuber in 1790, or that of the Joseph and Pierre Dufour brothers in the Burgundy region (France). Before 1830, wallpapers were made by painting, stenciling, engraving, or most often by printing with woodblocks in distemper colors on joined sheets of handmade paper. After this date, thanks to the development of engineering techniques, the modern continuous paper production for wallpapers started [2].

The techniques of execution, the decorations, and the materials used in the West were different from the oriental wallpapers [3]. However, the use of toxic pigments for their realization had been largely documented in all cases [4,5]. For this reason, the interest in the study of wallpapers has been also focused on the impact that these materials may have had on the health in the past and especially nowadays. Previous research underlined the health impact of considerable amount of toxic products containing lead (Pb), chromium (Cr), arsenic (As), and mercury (Hg) widely used as pigments for interior wall decoration [6,7,8]. Quite famous is the case of the manufacturer William Morris, who used green arsenic pigments [9]. However, the use of toxic paintings is not only something that happened in the past. In fact, the research of Mielke et al. [2] concerning the content of Hg and Pb in coatings of latex paint, underlines their risk mainly for the children who may ingest paint chips and consequently develop lead poisoning. Moreover, in 1977, the United States Environmental Protection Agency (EPA) banned Pb-based paints, and in 1991, the same agency banned Hg in the form of phenyl-mercury acetate, added as a preservative in house paints. Thus, technicians and restorers should be aware of the danger of the materials with which they come into contact in order to adopt adequate protective measures. 

As mentioned, in comparison with other type of artworks, wallpapers are easily degradable by intrinsic factors, due to the materials and techniques used, or extrinsic agents such as light, humidity, micro-organisms, etc. In fact, their fragility is strongly affected by the characteristics of the architectural wall and of the environment [10]. In addition, the constant changes in people’s taste to which they have been subjected over the years have facilitated their disappearance. We can almost consider them as ephemeral artworks depending on the trends of the moment [1,11].

Regarding the pigments, they can show degradation phenomena (chromatic change for example) caused by their natural aging or by the presence of contaminants in the environment. On the other hand, the cellulose matrix is the one that undergoes the greatest degradation due to reactions that can induce both chemical and physical changes. In most cases, the deterioration is irreversible, thus, it is important to understand the causes that could have provoked it. In particular, cellulose degradation occurs through the acid hydrolysis, oxidation, cross-linking, ring opening, chain scission, and variation of the amorphous/crystalline ratio [12], sometimes induced by the presence of some pigments (copper pigments) [13]. 

Due to the complexity in the composition of paper artworks, some authors have pointed out the usefulness of using many analytical techniques for the study of raw materials and for the investigation of the conservation state of the support [14,15,16]. For this aim, non-destructive portable systems were developed and successfully employed during in situ campaigns for elemental (energy dispersive X-ray fluorescence spectroscopy, scanning electron microscopy—energy dispersive X-ray spectroscopy, laser-induced breakdown spectroscopy) and molecular study (FTIR and Raman spectroscopy in different modes, surface-enhanced Raman spectroscopy) of cultural heritage materials [17]. In most of the cases, decorative wallpapers are made by a superimposition of various painting layers. For this reason, due to the penetration power of the beam, the interpretation of the results is critical since the spectra recorded, both with elemental and with molecular techniques, can give information corresponding to many layers. In this circumstance, no destructive benchtop instrument allows to select specific points and, in some cases, perform maps of the distribution of element and compounds, for a better characterization of materials and the study of the manufacturing technique [13,18].

In the current study, as part of the restoration works, a series of decorative wallpapers belonging to the nineteenth century were studied by non-destructive elemental and molecular analyses. At first, micro-energy dispersive X-ray fluorescence spectroscopy (micro-EDXRF) was carried out to assess the elemental composition of wallpaper samples. Then, Raman spectroscopy and attenuated total reflectance Fourier transform infrared spectroscopy (ATR-FTIR) measurements were performed with the aim to determine the materials used for the realization of wallpapers (pigments and binder media) and to assess the state of conservation of paper support. 

## 2. Materials and Methods

### 2.1. Historical Information and Samples

The samples under study were taken during the restoration works of Arizmendi Enea-Urdiñola Palace in Oiartzun (Basque Country, Spain) carried out to convert the building into a museum. The building, built in the 18th century, has maintained its actual appearance for more than three centuries and represents an example of the lifestyle of the more powerful classes of the province of Gipuzkoa in the 19th and 20th centuries. During the Spanish civil war, the Arizmendi Enea palace fell into the hands of the Francoist authorities where they established their headquarters. 

The wallpapers were made of vellum paper of industrial manufacture. As they are made of continuous paper, they do not come before 1830. The Zuber & Cie factory in Rexheim (Alsace, France) specialized in the manufacture of wallpapers, and managed to make the first continuous paper in its paper mill in 1830, obtaining a patent for it in 1832. However, no marks of the production company were found so their origin remains unknown. 

Decorative wallpaper can represent different themes depending of the period and provenance [19]. In the past, the design could show repetitive schemes of flowers and medallions in undulating arrangements [20]. In other cases, they imitated expensive material such as painted wall decoration, marble, ivory, and most often textiles with imitations of rich fabrics such as moirés and velvets [2]. The wallpapers studied in the present work belong to the latter design with a serial image. The execution technique was the glue tempera applied by means of xylographic stamping procedures. 

The analyzed samples were obtained from the decorative wallpapers after being removed from the palace and before being restored in the conservation and restoration laboratory of the Department of Painting of the University of the Basque Country (UPV/EHU). A total of five samples (wp1-wp2-wpP3-wp4-wp5) of different sizes were analyzed (the largest had a size of about 7 × 4.5 cm). They showed the main shades used for decoration including green, purple, black, white, grey, and brown colors. The state of conservation of wallpaper was quite good although some areas, in correspondence with the ceiling, presented a chromatic discoloration of the paper support. 

### 2.2. Instrumentation

#### 2.2.1. Micro-Energy Dispersive X-ray Fluorescence Spectroscopy (micro-EDXRF)

The elemental maps were acquired using a M4 TORNADO EDXRF spectrometer (Bruker Nano GmbH, Berlin, Germany). The analyses were performed under vacuum (20 mbar) in order to improve the identification of the lighter elements. The lateral resolution used for spectral acquisitions was 20 micrometers. The maps were obtained using M-QUANT software. To obtain the quantitative maps, the assignment of the elements and the deconvolution of the spectral information were carried out. The maps were obtained by considering the K-alpha line of each element. 

#### 2.2.2. Micro-Raman Spectroscopy 

Wallpaper samples were analyzed using a confocal Renishaw InVia Raman spectrometer, coupled to a Leica DMLM microscope. The spectra were acquired with the Leica 50× N Plan (0.75 NA) lens with a 2 μm spatial resolution and the Olympus 100× (0.8 NA). The minimum theoretical spot diameter using the 532 nm laser was, for the Leica 50× and Olympus 100×, 0.9 μm and 0.8 μm, respectively, while using the 785 nm laser, it was 1.7 μm and 1.1 μm, respectively. Additionally, for visualization and focusing, another Leica 5× N Plan (0.12 NA) and a 20× N Plan EPI (0.40 NA) lens were used. For focusing on and searching for points of interest, the microscope implements a motorized stage (XYZ). The power applied was set at the source at a maximum of 50 mW while on the sample was always less than 20 mW to avoid possible thermodecomposition of the samples. Normally, 10–300 scans, each lasting 1–20 s, were accumulated to achieve a suitable signal-to-noise ratio at an operating spectral resolution of ≤1 cm^−1^.

#### 2.2.3. Attenuated Total Reflection Infrared (ATR-FTIR)

ATR-FTIR spectra were collected by a Jasco 6300 spectrophotometer equipped for standard transmittance and attenuated total reflectance (PIKE MiracleTH) measurements. The equipment is featured by a Ge/KBr beam splitter and a deuterated L-alanine doped triglycene sulfate (DLATGS) detector with Peltier temperature control. All spectra were acquired in the middle infrared region (from 4000 to 400 cm^−1^), accumulating 128 scans, with a spectral resolution of 4 cm^−1^. Data acquisition was carried out by Jasco Spectra Manager Suit package, whilst data treatment was performed by Origin 2018.

#### 2.2.4. pH-Meter 

For the analysis of the pH of the cellulose a pH-meter (micropH 2000 Crison) was used. The electrode (52 07, Crison) for pH measurements on flat surfaces was employed. Its main characteristic is that its diaphragm and the membrane are on the same plane. 

## 3. Results and Discussion

### 3.1. Characterization of Materials

**Figure 1:**
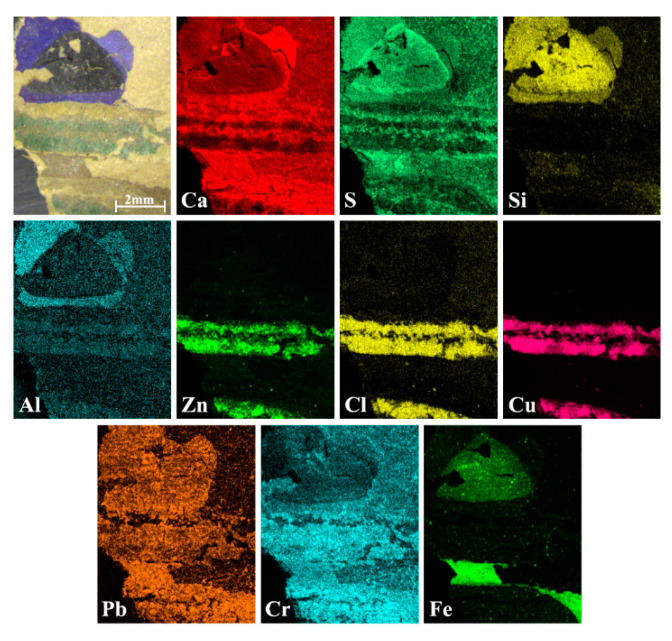
Micro-EDXRF maps from one of the decorative wallpaper samples (sample wp2).

The wallpapers samples were analyzed at first with micro-EDXRF to obtain the elemental distribution of the compounds employed for their realization. For this purpose, micro-EDXRF maps were recorded like shown in Figure 1. Although a total of five samples were analyzed, the selected areas shown in the elemental distribution maps of Figure 1 were representative of the whole analyzed samples as they included all colors of the wallpapers; green, brown, purple, black, grey, and white. The size of the sample was larger than that shown in the images. 

The most abundant elements identified in the first sample were calcium (Ca) and sulfur (S) (Figure 1) and their distribution was visible even where the paint layer was lost. In addition, Pb and Cr were distributed fairly evenly throughout the sample. Aluminum (Al) was present in well-defined areas where a purple pigment was applied, whereas silicon was in black and violet areas. For the green-colored areas, micro-EDXRF analysis showed the presence of copper (Cu), denoting the use of a green or blue copper pigment. In the same green areas, zinc and chlorine were also evident. On the other hand, in brown and black zones, iron (Fe) was found. 

After micro-EDXRF, Raman analyses were carried out to know the molecular composition of the raw materials and to identify any degradation process they may have undergone over the years. 

The use of calcite as well as gypsum was recognized in many points of analysis even where the painted layer was not present. They were applied as fillers of cellulose like reported in other investigations [14]. In particular, calcium carbonate was employed also like the alkaline reserve for the paper support to neutralize the acidic nature of some component of cellulose (lignin) and avoid its oxidation and acidic hydrolysis. These results are in concordance with the distribution of Ca in the EDXRF analysis (see above).

**Figure 2:**
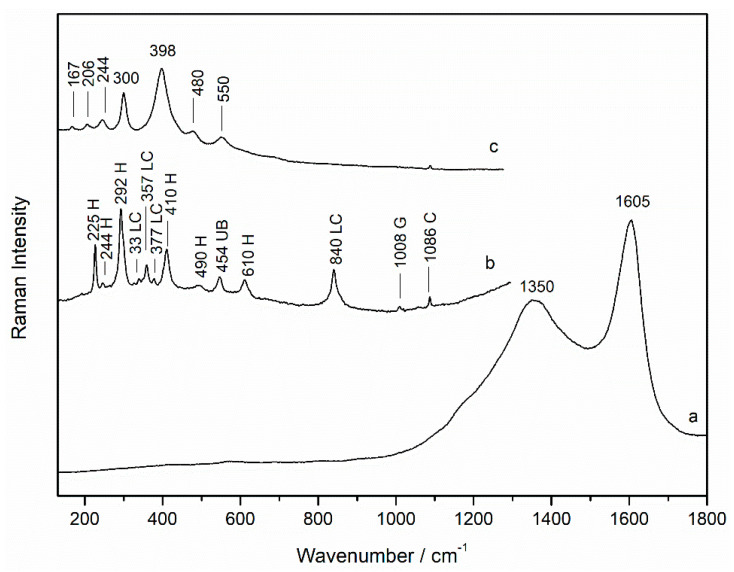
Raman spectra of (**a**) carbon black (532 nm excitation laser), (**b**) hematite together with the Raman bands of lead chromate (LC), ultramarine blue (UB), gypsum, and calcium carbonate, and (**c**) goethite (785 nm excitation laser) from a black area (sample wp2).

Raman spectra of the black areas showed the presence of carbon black pigment (Raman bands: 1350 and 1605 cm^−1^, Figure 2a). Iron oxides hematite (Raman bands: 225, 244, 292, 410, 490, and 610 cm^−1^, Figure 2b) and goethite (Raman bands: 167, 206, 244, 300, 398, 480, and 550 cm^−1^, Figure 2c) were also detected in black areas mixed with carbon black, as observed also with the optical microscope (red and yellow grains mixed with the black pigment). Their presence explains the evidence of iron found by micro-EDXRF in that area. 

**Figure 3:**
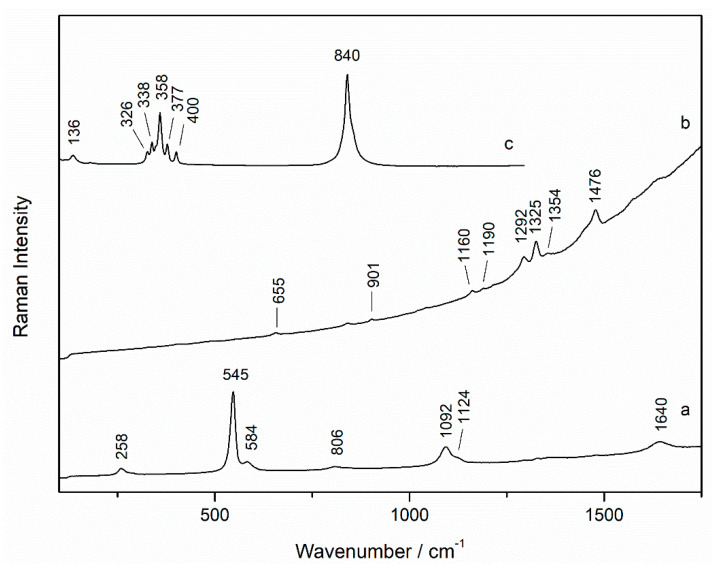
Raman spectra of (**a**) ultramarine blue, (**b**) alizarin red (532 nm excitation laser), and (**c**) lead chromate (785 nm excitation laser) from a purple area (sample wp2).

According to Raman spectroscopy, the purple shade was obtained by a mixture of ultramarine blue (Al_6_Na_8_O_24_S_3_Si_6_, Raman bands at 258, 545, 584, 806, 1092, 1124, 1290, and 1642 cm^−1^, (Figure 3a) and the synthetic organic pigment alizarin lake (PR 83:1) (Raman bands at 484, 655, 901, 1190, 1292, 1325, 1354, and 1476 cm^−1^, (Figure 3b) [21,22]. The unexpected presence of silicon (Si) detected in the black areas by micro-EDXRF would be explained by the use of ultramarine blue, since a purple layer was found below in that area. Here, we have an example of the problem explained above, how the superimposition of several layers can make difficult the interpretation of EDXRF spectra in this case. However, from the comparison of EDXRF maps of Al and Si, it was also evident that a silicon-based pigment was used in black areas in mixture with carbon black and iron oxides but it could not be identified by Raman spectroscopy. 

In addition, in most points analyzed and according to the distribution of lead and chrome (by EDXRF), the yellow pigment lead chromate (PbCrO_4_, Raman bands at 136, 326, 338, 358, 377, 400, and 840 cm^−1^, Figure 3c) was identified by Raman spectroscopy. This pigment was employed to obtain different colors in mixtures with other pigments. For example, in green areas, it was mixed with ultramarine blue. 

**Figure 4:**
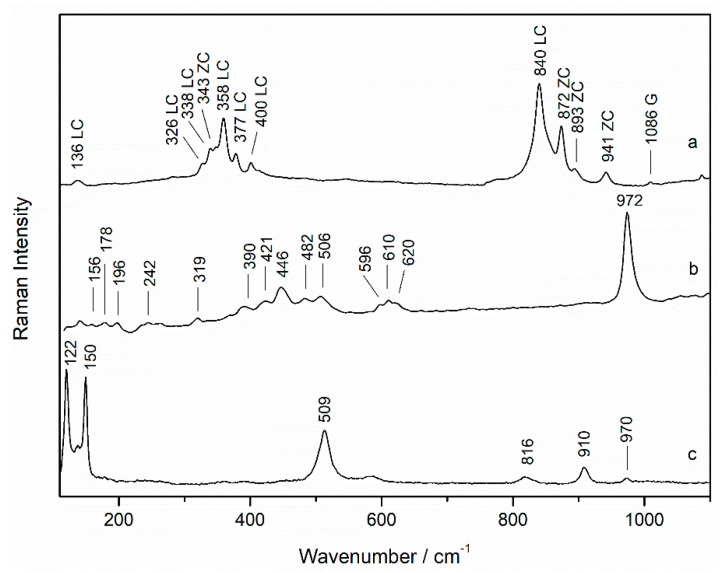
Raman spectra of (**a**) lead chromate (LC) in mixture with zinc chromate (ZC), (**b**) brochantite, and (**c**) atacamite (785 nm excitation laser) from a green area (sample wp2).

Additionally, the yellow pigment zinc chromate (ZnCrO_4_ Raman bands: 112, 141, 343, 358, 410, 774, 872, 893, and 941 cm^−1^, Figure 4a) was identified in the areas where zinc appeared, mixed with lead chromate and ultramarine blue in order to obtain the green color. On the other hand, in the same areas, in minor amount, two green pigments, the copper sulfate brochantite (Raman bands: 156, 178, 196, 242, 319, 390, 421, 446, 482, 506, 596, 610, 620, and 972 cm^−1^, Figure 4b) and the basic copper chloride atacamite (Cu_2_(OH)_3_Cl, Raman bands: 122, 150, 509, 816, 910, and 970 cm^−1^, Figure 4c) were identified, in concordance with the distribution of copper and chlorine in the EDXRF maps. Previous research mentioned atacamite, alone or together with brochantite, as a degradation product of copper pigments such as malachite or azurite, although its use as pigment has been also demonstrated [23,24]. In our case, no Raman spectra of malachite was recorded, thus, the use of atacamite mixed on purpose with brochantite was assumed. In the green areas, the presence of gypsum was also found in many points as well as carbon black. 

In the dark brown lines, where iron was detected by EDXRF, the presence of a mixture of lead chromate and hematite was found. In addition, gypsum as well as calcite were used on purpose as pigments, due to the distribution of calcium and sulfur in well-defined areas in the EDXRF maps. 

**Figure 5:**
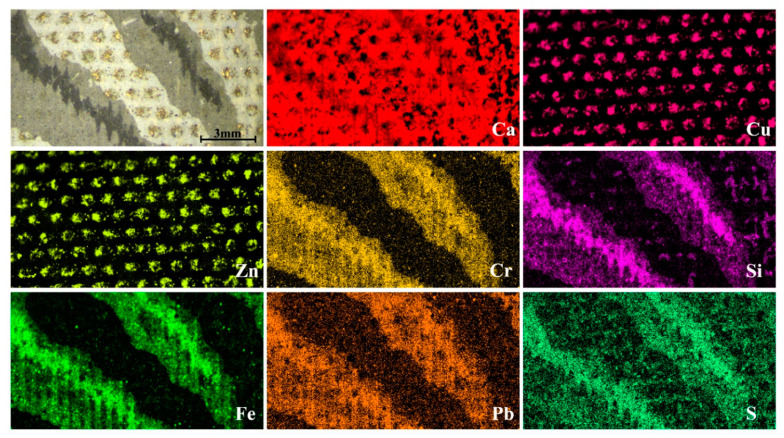
Micro-EDXRF maps from a decorative wallpaper (sample wp4). After the systematic application of brass all over the paper, other pigmented layers were applied.

The second analyzed sample was characterized by a circular bright decoration applied over a white and grey background with some black areas that simulate a textile material, like shown in Figure 5. EDXRF analyses showed that also in this case the most abundant element in the sample was calcium, distributed in a homogeneous way throughout the area. 

In addition, in this sample, the layered process of manufacturing the wallpaper was noticed thanks to the use of micro-EDXRF maps. Indeed, the metallic decoration, which was not evident in the entire sample by the naked eye, was clearly visible in the micro-EDXRF maps, and it was composed by copper (Cu) and zinc (Zn). As mentioned, wallpaper manufacture consisted in the superposition of various colored layers using blocks of wood on which the drawing was engraved. In our sample, at first, a white layer was applied above which the Zn and Cu decoration was spread. Subsequently, the grey layer and lastly, the black one, were applied according to the desired decoration. 

Even in these samples, according to the distribution of chrome and Cr, lead chromate was detected in many points regardless of the color. In the grey areas, it was employed together with ultramarine blue, calcite, and carbon black. On the other hand, in white areas, calcium carbonate (CaCO_3_, Raman bands: 155, 282, 712, and 1086 cm^−1^, Figure S2b) was mainly identified in mixture with gypsum (CaSO_4_·2H_2_O, Raman bands, 414, 494, 620, 670, 1008, 1135 cm^−1^, Figure S2a) and grains of lead chromate. In addition, the white pigment barium sulfate (BaSO_4_, Raman bands: 453, 460, 615, 645, 987, 1138, and 1164 cm^−1^, Figure S2c) was identified. In the black areas, where the presence of iron and silicon were found by micro-EDXRF, the carbon black was mixed with ultramarine blue and the yellow pigments with lead chromate and goethite. 

As already mentioned, some analyzed samples were characterized by the presence of a golden decoration in addition to black, white, and grey colors. The elemental composition obtained in a bright circular area and in a grey area is shown in Figure S3. Analyses carried out in different points showed the difference in the concentration of copper and zinc in the metallic circles. The high concentration of these elements suggested the presence of brass like decorative element due to its brightness and used as a substitute for gold. This decorative compound has also been identified in other studies carried out on wallpaper belonging to the nineteenth century [25]. According to the elemental maps, a binary brass was used since, in addition to Cu and Zn, no other metal, such as aluminum, manganese, or silicon was detected. However, in the EDXRF spectra recorded in the circular areas (Figure S3a), other elements (calcium, iron, lead, silicon, sulfur, chrome) were recorded, due to the penetration capability of the EDXRF technique, and coming from the paper support and painted layers. They were more evident in the spectra collected in a grey area (Figure S3b). A semi-quantitative analysis of the brass was carried out without considering the elements from underlying layers. The percentage of Zn and Cu was 29.4% and 70.6%, respectively, which was compatible with the compositional range of α-brass that has a zinc content of less than 35%. (α-β brasses have between 35% and 46.6% of Zn while β-brasses between 46.6% and 50.6% of Zn) [26]. The addition of zinc influences the corrosion resistance, which decreases if a major amount is added. As a result, a low brass zinc content will be often more resistant than brass with higher zinc grade [27]. The alpha brasses are ductile, much more when they are cold-worked and annealed rather than hot-worked because, if hot-worked, impurities tend to segregate at the grain boundaries and make the brass very weak [26].

In addition, to carry out a complete characterization of the materials, ATR-FTIR analyses were also performed. In all, ATR-FTIR spectra recorded on the back side of the paper support (Figure S4a) the presence of the peaks belonging to calcium carbonate, vibration modes were visible at 713 (ν_4_ CO_3_, symmetric), 873 (ν_2_ CO_3_, asymmetric), 1420 (ν_3_ CO_3_, asymmetric), and 1800 cm^−1^ (ν_1_ + ν_4_ CO_3_, symmetric) [28]. In addition, some bands were compatible with the fundamental stretching and bending vibrations observed in the infrared region for SO_4_^2−^ (ν_3_, 1115 and 1143 cm^−1^) and for H_2_O (ν_2_ 1620 cm^−1^, ν_1_ 3400, ν_3_ 3540 cm^−1^) of gypsum [29]. Thus, these results were in accordance with EDXRF maps confirming the use of calcite as alkaline charge of paper support and of the gypsum as filler of the cellulose. 

In addition, thanks to the FTIR analyses, the presence of an organic compound was found. Indeed, the band at 1642 cm^−1^ corresponds to a fundamental stretching of the amide carbonyl group (–CO–NH–, 1655 cm^−1^) and the deformation of amide II (–NH_2_, 1632 cm^−1^) [30]. In the FTIR spectrum methylene group, absorbencies at 2853 and 2923 cm^−1^ were also evident, and the broad band centered at about 3300 cm^−1^ (N-H stretching), indicating hydroxyl and amide groups partially masked by the broad absorption band of the OH group [31]. The evidence in the ATR-FTIR spectrum was compatible with the presence of animal glue. In addition, the FTIR bands belonging to the animal glue were evident in the spectrum recorded in a purple area (Figure S4b) showing the use of an organic binder for the application of pigments in the wallpapers. 

### 3.2. State of Conservation of the Wallpapers

The analytical techniques were applied to assess the state of conservation of paint layer as well as of the paper support. 

Regarding the state of conservation of pigments, a chromatic alteration of the pictorial layer, in the form of darkening, was visible. No degradation products of the original pigments were identified as responsible for the chromatic change by Raman or FTIR spectroscopy. The presence of carbon black, found in many points of the sample, seemed to be the main factor of discoloration of the wallpaper and it could be considered a contamination due to the presence of black particulate matters. However, due to the tendency of lead chromate to darken [32,33], caused by a reduction of the chromate ions to Cr(III)-compounds, its involvement in the degradation process cannot be excluded despite the degradation product (Cr_2_O_3_·2H_2_O) was never recorded by Raman spectroscopy in the studied wallpaper. Indeed, in the areas of brown color, a greater quantity of lead chromate was identified and at the microscopical level, they appeared dark yellow. Therefore, it is possible that those areas were yellow originally with a different shade depending on the pigment (hematite, goethite, gypsum, etc.) mixed with lead chromate.

Micro-EDXRF analyses were also performed in some areas, in the back side of the support, where the paper showed an evident chromatic change due probably to an oxidative process. The elemental maps suggest the presence of sulfate compounds in correspondence with the darker areas as shown by the overlapping of the sulfur, calcium, and barium maps (Figure S5). According to the Raman analyses performed in these areas, the main compounds identified were gypsum and barium sulfate. The evidence of barium sulfate and gypsum could be related with the presence of a paint layer spread on the walls before the application of the wallpapers. 

**Figure 6:**
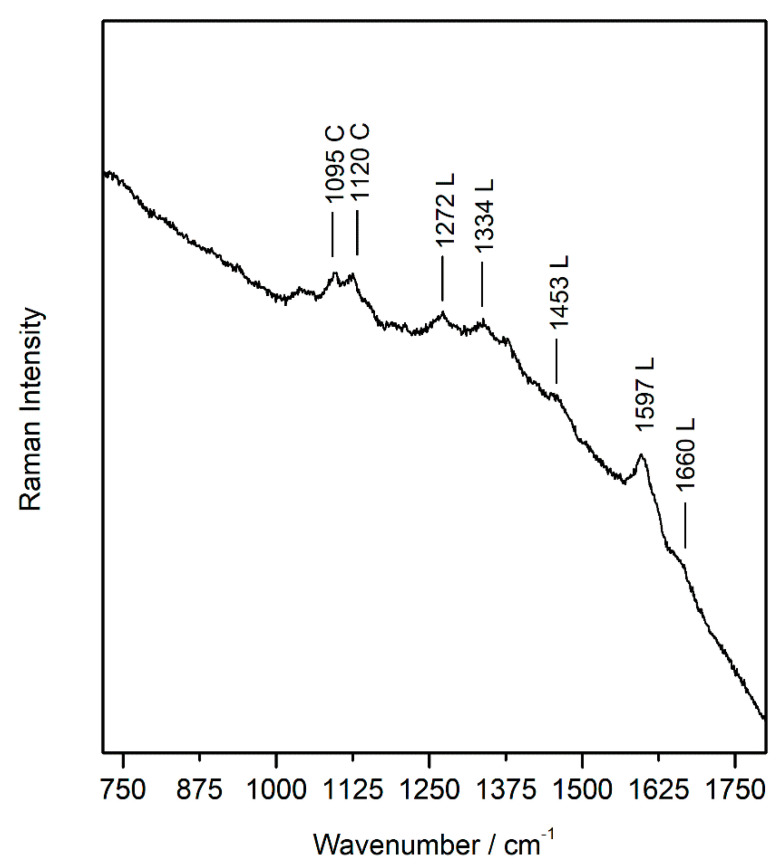
Raman spectrum of paper support where bands of cellulose (C) and lignin are shown (L) (sample wp5).

In the Raman spectra recorded in the same areas (Figure 6), the Raman bands of cellulose, the principal component of paper, were visible. Cellulose is composed by both crystalline and amorphous phases. The most abundant native crystalline form is cellulose I that exists as a mixture of polymorphs I_a_ and I_b_ [34]. In addition to cellulose, the paper matrix is composed in a minor amount by hemicellulose and lignin. Hemicellulose and the amorphous component of the cellulose act as a linker between the crystalline cellulose fibres [12]. Lignin is a heterogeneous polymer that occurs in woody and vascular tissues. It forms a matrix surrounding the cellulose in woody cell walls, which protects the hemicellulose and cellulose that together form the holocellulose [35].

In Figure 6, two bands belonging to cellulose were visible located at 1095 cm^−1^ (C–O–C of β-glycosidic linkage of cellulose and hemicellulose) and at 1120 cm^−1^ (C–O–C of the α-glycosidic linkage of hemicellulose). Generally, the aging processes of cellulose favor the partial breaking of the α-glycosidic and β-glycosidic. In particular, as demonstrated by previous research, a decrease in the intensity of the band corresponding to the α-glycosidic bond, compared to the β -glycosidic bond can occur, because it is a weaker link, therefore, the hemicellulose seems to be more sensitive to degradation processes [36]. 

In addition to the cellulose Raman bands, those belonging to lignin were evident, although very weak, with the most intense one at 1597 cm^−1^ corresponding to the symmetric aromatic ring stretch. All the Raman bands of lignin (1272, 1334, 1453, 1597, and 1660 cm^−1^) present in the Raman spectrum recorded in the samples have been reported in the study of Agarwal et al. [37] for the spectrum of spruce milled-wood lignin (MWL). The presence of lignin in the paper matrix could be one of the causes of the cellulose oxidation, due to its acidic nature, which was visible by the naked eye like a yellowing (Figure S5). For this reason, nowadays, lignin is removed from cellulosic pulp before the manufacturing process of paper. In addition, the absence of some Raman bands at 2893 and 2936 cm^−1^ could be attributed to the aging of cellulose as reported in another investigation [38]. In order to verify the pH of the paper support, some analyses with a pH-meter were carried out in the yellowed areas. Ten points were considered, and the average of the values obtained was between 4.23 and 5.57, in the most yellowed and in the best preserved areas, respectively, confirming an acid pH of the cellulose support. This fact suggested an important degradation process of cellulose underling the loss of effectiveness of the alkaline reserve. 

## 4. Conclusions

This work provides a successful application of complementary analytical techniques for the investigation of materials employed for the manufacturing of decorative wallpapers from the beginning of the 19th century. The proposed approach that included the use of non-destructive and non-contact elemental and molecular spectroscopic techniques was considered the most reliable for the study of cultural heritage materials. 

At first, micro-EDXRF images, employed mainly for the study of the elemental distribution of compounds, allowed us to identify the layered manufactured process of the wallpapers, showing even the painted layer not visible by the naked eye. In addition, this technique revealed the presence of Cu and Zn in bright points, indicating the use of α-brass as a decorative element. The molecular analysis of the pigments was successful carried out by means of Raman spectroscopy and it was compatible with another investigation about decorative wall painting of the 19th century. The massive presence of chromium pigments, mainly lead chromate but also zinc chromate, should be taken into consideration during the restoration works. Thus, restorers and conservators will take adequate measures to preserve their health based on the results of this investigation.

In addition to the characterization of pigments, the use of Raman spectroscopy allowed to assess the conservation state of the paper support. The pH values of the paper support showed a strong acidification of the cellulose, probably due to the natural degradation of its alkaline reserve. The presence of lignin also contributed to the degradation and oxidation process of cellulose. Thanks to the use of ATR-FTIR spectroscopy, the presence of animal glue used both for the preparation of support and as a medium was identified. 

Due to the historical and artistic interest, a restoration intervention of decorative wallpaper was planned. The data obtained in this study, using different spectroscopic techniques, will be of crucial importance for choosing the best conservation measures and for restoring the original chromatic palette of the decorative wallpapers. 

## Data Availability

Not applicable.

## Supplementary Materials

The following are available online at https://www.mdpi.com/article/10.3390/s21134416/s1, Figure S1: Details of wallpaper representing upholstery. Figure S2: Raman spectra of (a) gypsum, (b) calcite plus gypsum (G) and (c) barium sulfate (785 nm excitation laser) from a white area (samples wp4). Figure S3: Micro-EDXRF spectra collected in a circular area (a) and in a grey area (b) (sample wp4). Figure S4: ATR-FTIR spectrum taken from the backside of decorative wallpapers (a) and from a purple area (b) (sample wp3). Figure S5: Micro-EDXRF maps from the degraded paper support (sample wp5).
