# Supplementary material for: Non-Destructive Analytical Investigation of Decorative Wallpapers Samples of the Nineteenth Century before Their Restoration
Source: Sensors (Basel). 2021 Jun 28;21(13):4416. doi: 10.3390/s21134416 (PMC8272152; doi:10.3390/s21134416)
Supplement: Supplementary file 1 [file sensors-21-04416-s001.zip › sensors-1263061-Supplementary.pdf]

# Supplementary Figures

# Non-destructive analytical investigation of decorative wallpapers samples of the nineteenth century before their restoration.

Ilaria Costantini[1], Kepa Castro[1], Maria Dolores Rodriguez-Laso[2], Juan Manuel Madariaga[1], Gorka Arana[1].

[1] Department of Analytical Chemistry, Faculty of Science and Technology, University of the Basque Country UPV/EHU, P.O. Box 644, 48080 Bilbao, Basque Country, Spain.
E-mail: ilaria.costantini@ehu.eus
2 Department of Painting, University of the Basque Country, P.O. Box 644, 48080 Bilbao, Spain

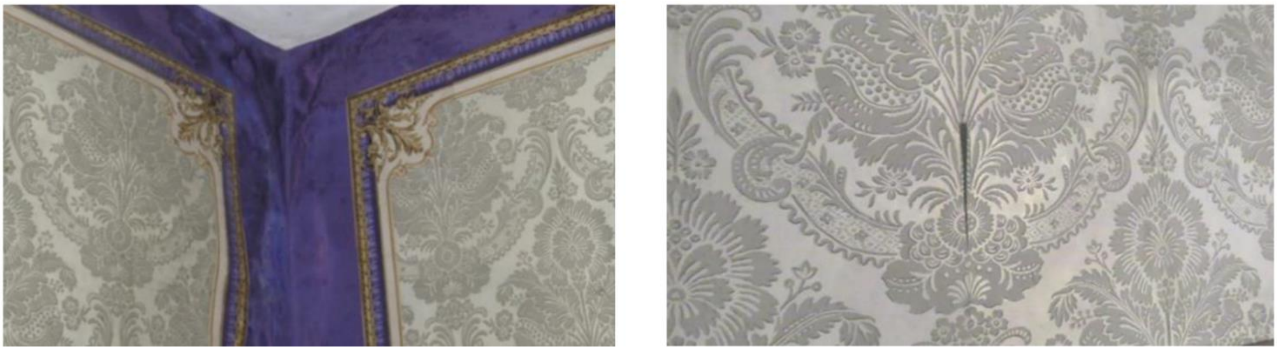

**Figure S1.** Details of wallpaper representing upholstery.

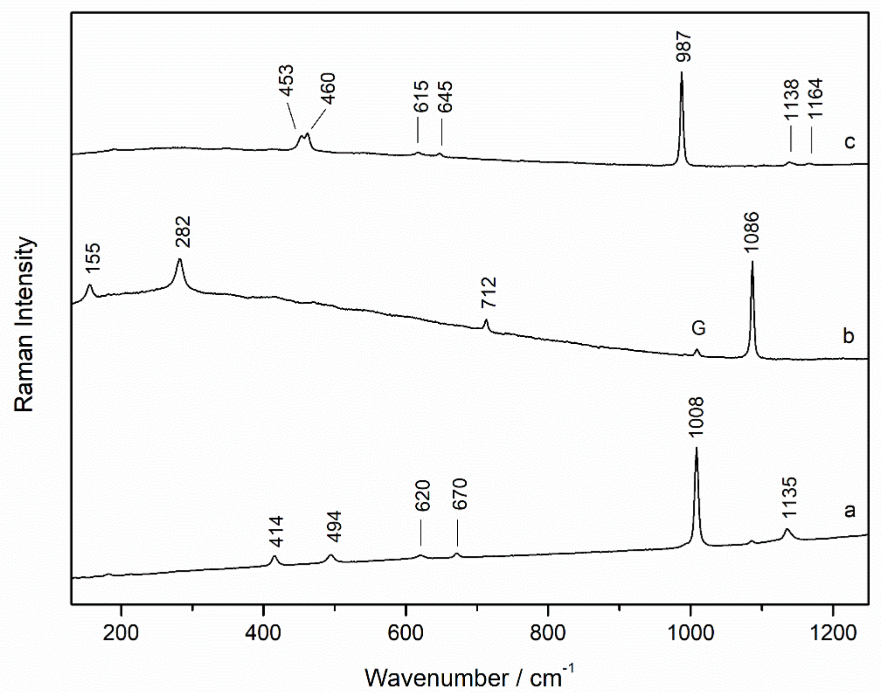


**Figure S2.** Raman spectra of (a) gypsum, (b) calcite plus gypsum (G) and (c) barium sulfate (785 nm excitation laser) from a white area (samples wp4).

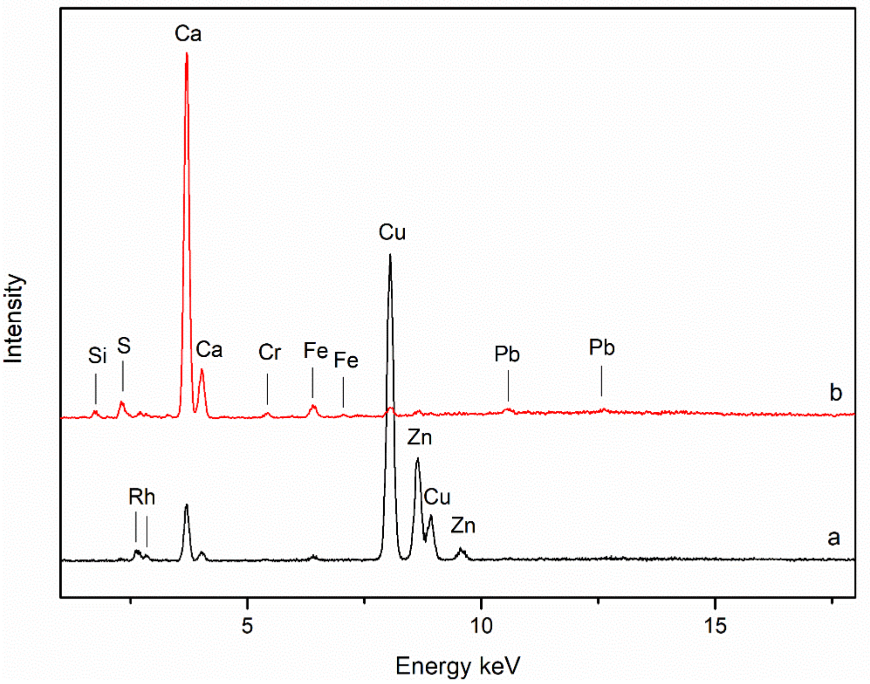


**Figure S3.** Micro-EDXRF spectra collected in a circular area (a) and in a grey area (b) (sample wp4).

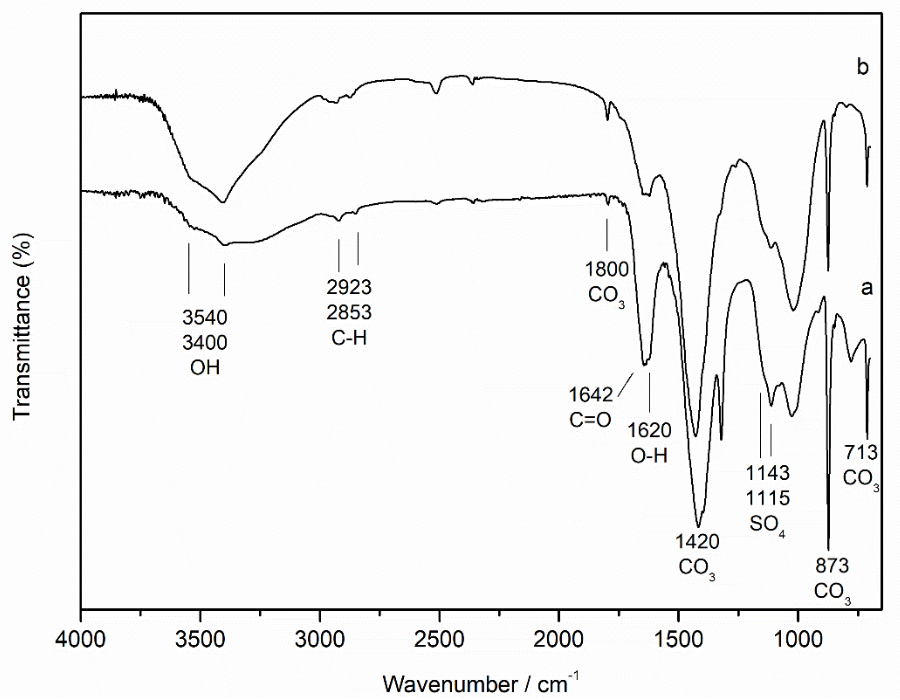


**Figure S4.** ATR-FTIR spectrum taken from the back side of decorative wallpapers (a) and from a purple area (b) (sample wp3).

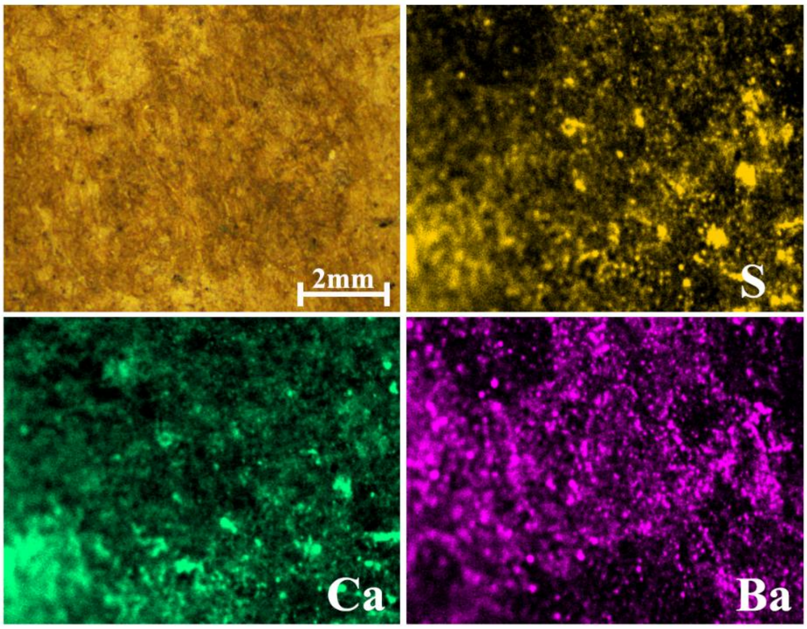


**Figure S5.** Micro-EDXRF maps from the degraded paper support (sample wp5).
